# CD64 binding potential does not translate into enhanced therapeutic efficacy for anti-IL-23 antibodies under physiologically relevant conditions

**DOI:** 10.1186/s10020-026-01462-z

**Published:** 2026-03-28

**Authors:** Joel F. Cohen-Solal, Calvin S. Pohl, Sheila M. Cummings, Jeremy P. Gygi, Zhaleh Safikhani, Brigitte Bartocha, Christopher D. Buckley, Yongli Dong, Killian Eyerich, Samuel D. Karsen, Grace R. Lynch, Michael Macoritto, Pierre A. Morisset, Ornella D. Nelson, Timothy Radstake, Florian Rieder, Jocelyn Rivas, John P. Savaryn, Kathleen M. Smith, Madison Stulir, Carmin Szynal, Casey Tylek, Geertruida M. Veldman, Laura G. Wasserman, Susan Westmoreland, Neha Chaudhary, Matthew M. Staron

**Affiliations:** 1 AbbVie Inc. Bioresearch Center, 100 Research Drive, Worcester, MA 01605 USA; 2 Kennedy Institute of Rheumatology, University of Oxford, Oxford, UK; 3 Translational Gastroenterology & Liver Unit, John Radcliffe Hospital, Headington, Oxford UK; 4 Rheumatology Research Group, Institute of Inflammation and Ageing, University of Birmingham, Birmingham, UK; 5 NIHR Oxford Biomedical Research Centre, Oxford, UK; 6 Department of Dermatology and Venereology, Medical Center, University of Freiburg, Freiburg, Germany; 7 Division of Dermatology and Venereology, Department of Medicine Solna, Karolinska Institute, Stockholm, Sweden; 8 AbbVie Inc., North Chicago, IL USA; 9 Department of Inflammation and Immunity, Cleveland Clinic Research, Department of Gastroenterology, Hepatology and Nutrition, Digestive Diseases Institute, Cleveland Clinic Foundation, Cleveland, OH USA

**Keywords:** CD64, Crohn’s disease, Fc portion, FcγRI, Guselkumab, Inflammatory bowel disease, Psoriasis, Risankizumab, Ulcerative colitis, Ustekinumab

## Abstract

**Background:**

Interleukin (IL)-23 neutralizing antibodies are clinically efficacious but differ in the functionality of their Fc portion. Guselkumab (GUS) and ustekinumab (UST) have a wild-type (WT) Fc portion, permitting native binding to Fc gamma receptors (FcγRs), while risankizumab (RZB) lacks FcγR binding due to the L234A to L235A (LALA) mutation. Recently, GUS was reported to neutralize IL-23 more effectively than RZB in vitro, owing to GUS-mediated binding of FcγRI (CD64) on macrophages. However, these findings do not account for the fact that FcγRI (CD64) is competitively occupied by endogenous immunoglobulin (Ig)Gs in vivo, as it is a high-affinity receptor for monomeric IgGs.

**Methods:**

To investigate the contribution of the LALA mutation in the Fc portion to IL-23 neutralization in vivo, we administered anti-IL-23 antibodies bearing either WT or LALA-modified Fc portions to Ig-competent (*Il10*^*−/−*^) and Ig-deficient (*Rag2*^*−/−*^) preclinical colitis mouse models. Building on these in vivo experiments, physiological competition between therapeutic antibodies and endogenous IgGs on FcγRI (CD64) binding was next assessed using in vitro binding assays. Specifically, GUS, RZB, and UST antibodies were incubated with CHOK1 cells expressing FcγRI (CD64) or with activated primary human monocytes (CD64^high^) in RPMI medium, in the presence or absence of plasma. To understand if IL-23 is exclusively produced by cells that may bind monomeric, therapeutic IgG, single-cell RNA sequencing (scRNAseq) and spatial transcriptomic analysis were used to evaluate the overlap of *IL23A* and *FCGR1A (CD64)* transcripts in cells isolated from the skin of patients with psoriasis (PsO) and intestine of patients with inflammatory bowel disease (IBD), respectively.

**Results:**

In the *Il10*^*−/−*^ and *Rag2*^*−/−*^ murine models, treatment with anti-mouse IL-23 WT or LALA IgG2a mouse monoclonal antibodies similarly suppressed proinflammatory gene expression and inflammatory cell infiltration. In vitro assays using activated primary human monocytes (FcγRI^high^ [CD64^high^]) showed that GUS, UST, and control human IgG1 bound to FcγRI (CD64), whereas RZB did not. However, the presence of plasma inhibited the binding of all therapeutic antibodies to FcγRI (CD64). We confirmed that endogenous IgGs in plasma saturated inflammatory macrophages FcγRI (CD64). Finally, in patient samples, the *IL23A* transcript was not exclusive to *FCGR1A*^+^ *(CD64*^+^*)* cells.

**Conclusions:**

These data support the notion that IL-23 is efficiently neutralized by anti-IL-23 monoclonal antibodies, independent of Fc-mediated binding to FcγRI (CD64), under physiologically relevant conditions. Additionally, coexpression of *IL-23A* and *FCGR1A* was rarely observed in tissues from patients with PsO or IBD.

**Graphical Abstract:**

The journal encourages graphical abstracts, which will appear on the *Molecular Medicine* website alongside the published article.
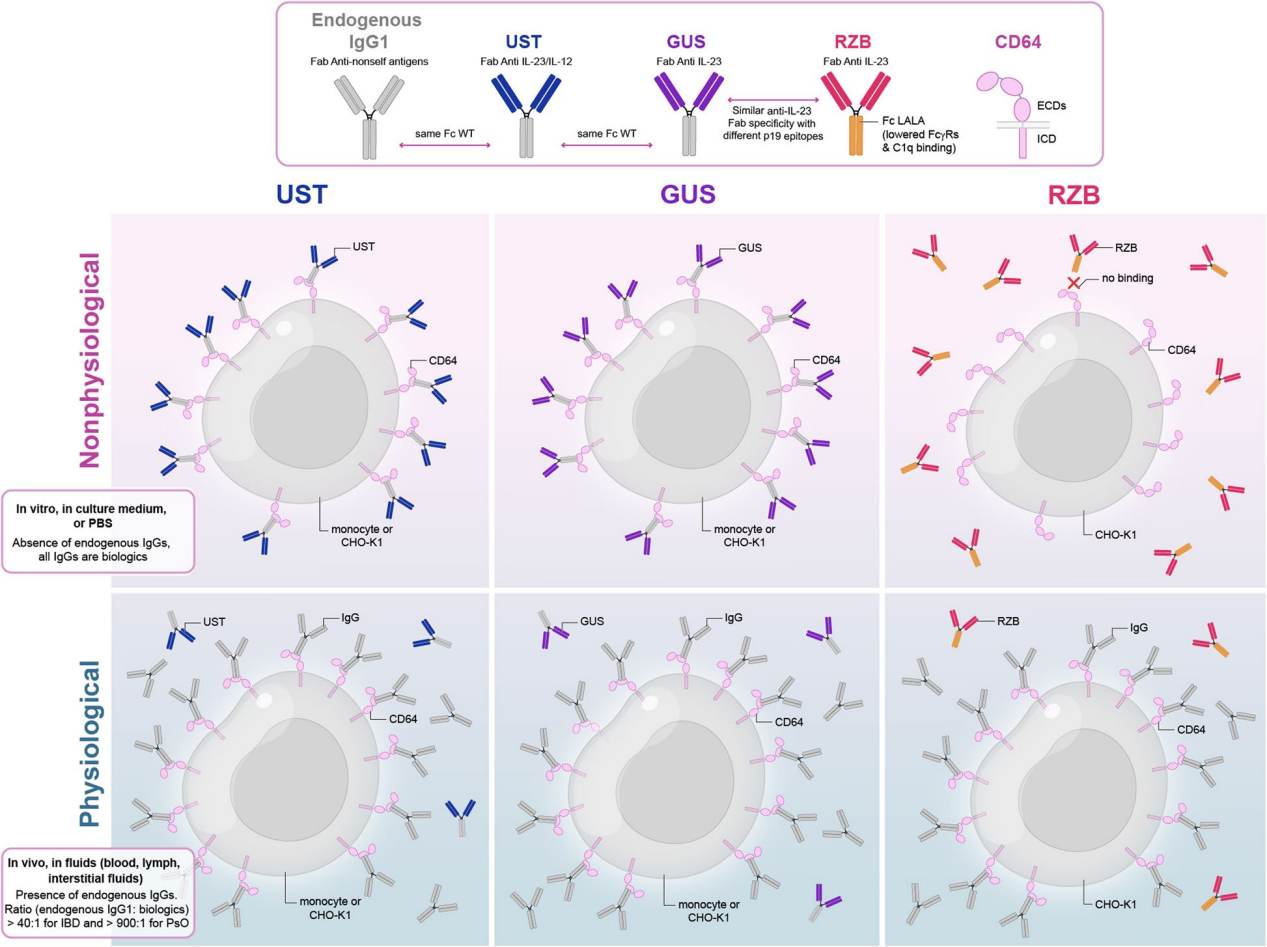

**Supplementary Information:**

The online version contains supplementary material available at 10.1186/s10020-026-01462-z.

## Introduction

Interleukin-23 (IL-23) is a proinflammatory cytokine implicated in the pathogenesis of multiple immune-mediated diseases, including inflammatory bowel disease (IBD), such as Crohn’s disease (CD) and ulcerative colitis (UC), as well as other inflammatory conditions such as psoriasis (PsO) (Moschen et al. 2019). IL-23 belongs to the IL-12 family of cytokines and is expressed in inflamed tissues by inflammatory monocytes, macrophages, dendritic cells (DCs), and neutrophils (Bourgonje et al. 2025). Upregulation of IL-23 promotes the production of IL-17A and IL-17F by innate and adaptive immune cells, leading to tissue neutrophil recruitment and tissue injury (Jairath et al. 2024).

The use of monoclonal antibodies (mAbs) to neutralize IL-23 signaling has revolutionized the treatment of immune-mediated diseases. IL-23 is a heterodimeric cytokine composed of the IL-23p19 (*IL-23A*) subunit and the IL-12p40 subunit (Moschen et al. 2019). Risankizumab (RZB) and guselkumab (GUS) are high-affinity, humanized immunoglobulin gamma 1 (IgG1) mAbs that selectively bind to IL-23p19 with high affinity and potency and prevent IL-23 from binding to its receptor (Jairath et al. 2024). Ustekinumab (UST) selectively binds to the IL-12p40 subunit and prevents both IL-23 and IL-12 from binding to their respective receptors; however, UST has lower affinity and potency than GUS and RZB for IL-23 neutralization and signaling inhibition (Zhou et al. 2021). Additionally, GUS and UST have functional Fc portions, similar to endogenous IgG1, allowing for potential binding to FcγRs and complement C1q. In comparison, RZB has an Fc portion with L234A to L235A (LALA) mutations, which reduces its binding to FcγRs and complement C1q (Singh et al. 2015; Hale 2024) RZB, GUS, and UST are approved for the treatment of UC, CD, PsO, and psoriatic arthritis (PsA) (Johnson and Johnson n.d.a, b, c, 2025; AbbVie, Inc. n.d.a, b).

A recent in vitro experiment demonstrated that GUS exhibits enhanced IL-23-neutralizing potency by directly binding to FcγRI (CD64) on IL-23-producing macrophages (Sachen et al. 2025). However, it is unclear if these in vitro findings could translate to enhanced clinical outcomes, given that the cellular source of pathogenic IL-23 may not be exclusive to FcγRI^+^ (CD64^+^) cells and that FcγRI (CD64) is saturated with endogenous monomeric IgG in vivo. Importantly, UST (wild-type [WT] Fc WT) and RZB (LALA Fc) have been compared head-to-head in PsO and CD, with RZB demonstrating superior clinical benefits (Peyrin-Biroulet et al. 2024; Gordon et al. 2018). Results from the UST and RZB head-to-head trials suggest that the interaction between anti-IL-23 antibodies and FcγRI (CD64) may not be a determinant of clinical efficacy.

In this study, we generated anti-mouse IL-23 mAbs with WT and LALA-modified Fc portions and compared their ability to neutralize IL-23-mediated inflammation in preclinical mouse models of colitis. In these models, IL-23 neutralization was independent of Fc-mediated binding to FcγRI (CD64). Differences in the mechanism of action were observed in the *Rag2*^*−/−*^ model that were not observed in the Ig-competent *Il10*^*−/−*^ mice, suggesting endogenous antibodies negate potential differences in IL-23 neutralization between antibodies with differences in the Fc portion, due to the presence or absence of the LALA mutation. To understand the lack of differences between anti-IL-23 antibodies in Ig-competent mice, we further tested in vitro whether WT Fc antibodies bind FcγRI (CD64) at physiological plasma concentrations. To understand the potential for WT Fc anti-IL23 antibodies to target IL-23-producing cells, we also assessed whether *IL23A* was produced exclusively by FcγRI^+^ (CD64^+^) cells in both PsO and CD single-cell RNA sequencing (scRNAseq) datasets. The lack of differences between the mAbs may be attributed to endogenous Ig competing with the WT Fc anti-IL-23 antibody, which binds to FcγRI^+^ (CD64^+^) cells, and the lack of exclusive expression of IL-23 by FcγRI^+^ (CD64^+^) cells.

## Methods

### Mice

*Il10*^*−/−*^ (B6.129P2-Il10tm1Cgn/J) and C57BL/6J WT mice were purchased from Jackson Laboratories (Bar Harbor, ME, USA); *Rag2*^*−/−*^ (B6.129S6-RAG2 < tm1Fwa > N12) mice were purchased from Taconic Biosciences, Inc. (Germantown, NY, USA). For all experiments, female mice, 6–8 weeks old, were used and maintained on a 12:12-h light: dark cycle. All animals were housed in specific pathogen–free conditions, with 5 mice per cage, and animal procedures were approved by the AbbVie Institutional Animal Care and Use Committee as outlined in the “Guide for the Care and Use of Laboratory Animals,” published by the National Academy Press (Academies. NRCotN 2011).

All animal procedures were conducted in accordance with institutional guidelines and approved by the Institutional Animal Care and Use Committee (IACUC) of AbbVie, in compliance with applicable national and international regulations for the care and use of laboratory animals. Efforts were made to minimize animal suffering and reduce the number of animals used wherever possible.

### Mouse models of colitis

To induce colitis in the *Rag*2^−/−^ mice, the animals received an intraperitoneal (ip) injection of anti-CD40 (clone FGK4.5; BioXCell, Lebanon, NH, USA) at a dose of 5 mg/kg (0.2 mL) in sterile 1 × Dulbecco’s phosphate-buffered saline (DPBS, Ca2^+^/Mg2^+^ free) on day 0. Anti-IL-23p19-mouse IgG2a WT Fc (AbbVie Bioresearch Center, Worcester, MA, USA; 45 mg/kg, 0.2 mL ip injection), anti-IL-23p19-mouse IgG2a L234A, L235A Fc (AbbVie Bioresearch Center; 45 mg/kg, 0.2 mL ip injection), or 1 × DPBS (Ca2^+^/Mg2^+^ free; 0.2 mL, vehicle control, ip injection) were administered on day −1 and day 3 (See Table S1 for additional information on anti-IL-23 mAbs). Mice were monitored, and their body weights were evaluated for 7 days. To induce colitis in *Il10*^*−/−*^ mice, drinking water containing 3% dextran sodium sulfate (DSS; MP Biomedicals, Santa Ana, CA, USA) was placed in the cage on day 0 and remained unchanged for 5 consecutive days. After day 5, the DSS water was replaced with fresh drinking water to allow the mice to recover. IL-23p19-mouse IgG2a WT Fc (anti-muIL-23 WT) or LALA-modified (anti-muIL-23 LALA) mAb were administered in sterile 1 × DPBS (Ca2^+^/Mg2^+^ free, 45 mg/kg in 0.2 mL) three times a week, on days 10, 14, and 17. A hydrogel (ClearH_2_O; Cincinnati, OH, USA) was provided to the mice daily from day 5 to day 11 as an additional source of hydration. Mice were monitored, and their body weight was evaluated for 21 days.

### Assessment of the inflammatory response in mouse models of colitis

To measure colitis and the associated inflammatory response, tissues (colon and blood) were harvested on either day 7 (*Rag2*^*−/−*^ mice) or day 21 (*Il10*^*−/−*^ mice), with lethal isoflurane used for euthanasia. Cardiac puncture was used to collect peripheral blood postmortem, with serum or plasma isolated for analysis of cytokines and chemokines, as well as exposure to anti-IL-23 mAb. Anti-IL-23 mAb and cytokines/chemokines were quantified in mouse serum or plasma samples using a total mesoscale discovery (TMD) singleplex assay with electrochemiluminescence detection employed (see Supplementary Methods for the protocol).

For gene expression analysis, the colon was removed, flushed with 1 × DPBS, and a 1 cm segment of the proximal colon was stored in RNALater (Invitrogen, Thermo Fisher Scientific) until processing for real-time quantitative polymerase chain reaction (qPCR). RNA isolation was performed using the RNeasy Fibrous Tissue Mini Kit with DNase I digestion (Qiagen, Germantown, MD, USA), followed by bead-milled homogenization. cDNA was synthesized using the High-Capacity cDNA Archive Kit (Applied Biosystems, Thermo Fisher Scientific), with RNA produced using the Taqman system (Invitrogen, Thermo Fisher Scientific). Genes assayed included *Il17f, Il6, Il22, Fcgr1, colony-stimulating factor 2 (csf-2), regenerating islet-derived-3β (Reg3β), tumor necrosis factor (Tnf), S100 calcium-binding protein a8* (*S100a8*), and *S100a9*, using gene-specific Taqman probes, with relative expression normalized to *Gapdh* or *β-actin*.

For immunohistochemistry (IHC) analysis, the colon was excised and flushed with 1 × DPBS (Ca2^+^/Mg2^+^ free) and 10% neutral buffered formalin. A piece of the distal/midcolon and anus (2 cm by 2.5 cm) was embedded and stored at −80 °C until sectioning and staining with a rabbit polyclonal ionized calcium-binding adapter molecule-1 (IBA-1) antibody (Wako Chemicals, Richmond, VA, USA). IBA-1 expression was quantitatively assessed in the proximal colon following 3,3'-diaminobenzidine (DAB) and hematoxylin staining. Quantification was performed using two Visiopharm applications. The first application, analogous to the mouse hematoxylin and eosin (H&E) neutrophil analysis module, was employed to generate a tissue region of interest (ROI). Within this tissue mask, IBA-1-positive areas were classified by applying an intensity threshold of 0–125 to the color-deconvoluted channel (HDAB–DAB, Visiopharm) at 20 × magnification.

For quantification of colonic mouse monocytes/macrophages, a single-cell suspension was isolated and prepared without formalin flushing; cells were stained with anti-CD45 BUV395 (clone 30-F11; BD Biosciences, Franklin Lakes, NJ, USA), anti-CD11b PE-Cy7 (clone M1/70; Biolegend, San Diego, CA, USA), anti-FcγRI APC (clone X54-5/7.1; Biolegend), and the Live/Dead Fixable Blue Dead Cell Stain (Invitrogen, Thermo Fisher Scientific). Flow cytometry data were collected using the LSR-Fortessa (BD Biosciences), and analysis was performed using FlowJo 10.7.2 software (Tree Star, Inc., Ashland, OR, USA).

### Quantification of mouse neutrophils

Neutrophil quantification was performed on H&E staining of the distal/midcolon. Two Visiopharm applications were used to quantify the percentage of neutrophils per total number of colonic cells. Whole-slide images (8-bit RGB) were first converted to a single grayscale channel (Visiopharm ISH-I) and median-filtered (5 × 5 pixel) to smooth tissue edges. Images were acquired at 0.5 × magnification. To distinguish tissue from background, a grayscale threshold of 230 to 255 was applied. Small artifacts (defined as areas < 1 × 10^6^ µm^2^) were eliminated using a ‘Change by Shape’ filter. The ‘Fill Holes’ function was subsequently used to restore integrity to isolated tissue islands. Within that mask, cell segmentation and classification applied a U-Net model (Visiopharm 2020.01.1.7332) fine-tuned on 75 tiles (1024 × 1024 pixels at 20 × magnification), with 20 reserved for validation; active learning in 25-tile increments (three iterations) with pathologist corrections yielded an intersection-over-union RN of 0.885 and an F1 score of 0.842 for neutrophil identification. To improve cell instance separation, pixel-wise probability maps from the segmentation probability maps were segmented into two labels: boundary (0.09–0.70) and interior (0.70–1.00). Subsequent postprocessing included watershed separation leveraging interior seeds and boundary objects. A secondary “Separate Objects” segmentation with a 7 µm diameter to further resolve clumps. Cells were then identified using “Change By Intensity” neutrophils by 0.4 −1.0.

### Bulk-RNA sequencing analysis

The Fastq files for this study were preprocessed using the `be_bulk_rnaseq` pipeline (version 2.2.0), developed by the AbbVie Bioinformatics Team. The Fastq files were initially processed using trim galore (v0.6.7). Genome references were sourced from Ensembl and mapped to Mus musculus using STAR (v2.7.11a). This pipeline utilized multiple open-source tools to perform quality control alignment and generate matrix counts using FeatureCounts (v2.0.3).

### Differential gene expression analysis

The differential gene expression (DGE) analysis for this study was conducted using the Bioconductor environment in R (v4.3.1). Initially, raw counts from the Feature Counts output were preprocessed and annotated using the ‘org.Mm.eg.db`(v3.18.0) package to link Ensembl IDs with gene symbols. Quality control metrics from various sources, including Picard, RNA-Seqc, and FASTQC, were generated and utilized to ensure sample integrity. After quality control, outliers detected using principal component analysis (PCA) in R (version 2.14.0) were discarded.

The count matrix was normalized using the Trimmed Mean of M-values (TMM) method and then transformed by a logarithmic function (Robinson and Oshlack 2010). The differentially expressed genes for each comparison were identified using the Limma method, with fold change > 2 and false discovery rate < 0.05 (Ritchie et al. 2015). Multiple contrasts were defined to compare the various experimental groups based on treatment conditions.

### Gene correlation analysis

To assess gene expression relationships in *Il10*^*−/−*^ mice treated with anti-mouse IL-23 mAbs with a WT or LALA-modified Fc portion, correlation analysis was performed with comparisons made against the 1 × DPBS-vehicle control group. The analysis focused exclusively on genes that exhibited significant differential expression (false discovery rate < 0.05) in at least one of the treatment comparisons.

Scatter plots were generated to visualize the log-fold changes in expression for these significant genes, with Pearson’s correlation and regression analyses to assess the strength and direction of the correlations. This analysis enabled the identification of gene expression patterns that are consistently or differentially modulated by the treatments, thereby offering insights into their respective molecular impacts. The visualization performed using the ggpubr (v0.6.0) package in R underscored significant correlations, providing a basis for interpreting the mechanisms underlying the observed gene expression changes (R Foundation and for Statistical Computing n.d.).

### Antibody binding assays

Chinese hamster ovary K1 (CHOK1), CHOK1 (humanFcγRI) and CHOK1 (mouse FcγRI) cell lines (Pasteur Institute, Paris, France) were cultured in Roswell Park Memorial Institute (RPMI) medium, 10% ultra-low IgG heat-inactivated fetal bovine serum (FBS), 1% NEAA (Gibco, Thermo Fisher Scientific, Waltham, MA, USA), and supplemented or not with G418 (Roche, Basel, Switzerland) and Zeocin (Invitrogen, Thermo Fisher Scientific). To express FcγRI (CD64), cell lines were transfected with two vectors: one conferring G418 resistance and expressing the human *FCGR1A* gene, and another conferring Zeocin resistance and expressing the mouse *Fcgr1* gene (Bruhns et al. 2009). Cells were stained with anti-human FcγRI (CD64, Allophycocyanin [APC] clone REA975, Miltenyi Biotec, Gaithersburg, MD, USA) or anti-mouse FcγRI (CD64 Alexa Fluor 647 clone X54-5/7.1, Biolegend San Diego,CA, USA) and analyzed by flow cytometry. RZB (human-IgG1 L234A, L235A), GUS (human-IgG1), UST (human-IgG1), control IgG1 (control human-IgG1), and mouse IgG2a anti-mouse IL-23 Fc WT or L234A, L235A mutated were produced internally and labeled with Alexa Fluor 647 (AF647; Invitrogen, Thermo Fisher Scientific) at 0 to 1 Alexa Fluor per IgG molecule for human IgG1s and 1–2 Alexa Fluor per IgG for mouse IgG2a; characterization was confirmed using mass spectrometry, evaluated by analytical size (data not shown) (Zhou et al. 2021).

GUS, RZB, and UST (in one group) and anti-mouse-IL23 surrogates (Fc WT or LALA mutated, in another group), both labeled with AF647, were incubated with CHOK1(NT), CHOK1(hu-*FCGR1A*), or CHOK1(mu-*Fcgr1*) cells. Cells were barcoded with either Cell Trace Carboxyfluorescein Succinimidyl Ester (CFSE) or Cell Trace Violet (Molecular Probe, Thermo Fisher Scientific) and mixed with either full human plasma, full C57Bl/6 mouse plasma, undiluted *Rag2*^*−/−*^ mouse plasma, human plasma diluted in binding buffer (RPMI + 10% ultra-low IgG heat-inactivated FBS (Gibco, Thermo Fisher Scientific), or in binding buffer alone for 60 min at 4 °C. For the diluted plasma experiment, a mix of 99.8% volume pure plasma (or binding buffer alone as GUS without plasma control) with 0.25% volume GUS (corresponding to a molecule ratio of 1:70, GUS to endogenous IgG1) was subjected to a two-fold serial dilution; each fraction was incubated with the barcoded cells. After a 1 h incubation at 4 °C, cells were washed three times with 1 × DPBS (Dulbecco’s phosphate-buffered saline; Ca2^+^/Mg2^+^ free, Gibco, Thermo Fisher Scientific), resuspended in 1 × DPBS, and analyzed by flow cytometry using the LSR-Fortessa (BD Biosciences). Cells were phenotyped for FcγRI (CD64) expression using the anti-FcγRI APC antibody (clone REA975, Miltenyi Biotec) on the LSR-Fortessa (BD Biosciences).

### Isolation and activation of human FcγRI^high^ (CD64^high^) monocytes

Peripheral blood mononuclear cells (PBMCs) from four healthy donors were isolated by Ficoll-paque plus (Cytiva, Marlborough, MA, USA) gradient, and monocytes were enriched by plastic adhesion in a flask for 12 h in RPMI, 10% FBS (Gibco, Thermo Fisher Scientific). Adherent cells were further incubated with 50 ng/mL of human-Interferon-γ (IFN-γ; InvivoGen, San Diego, USA) for 36 h. Adherent cells were recovered by incubation with Versene Gibco, Thermo Fisher Scientific) and phenotyped with anti-CD14 BV510 (clone M5E2, BD Biosciences) and anti-FcγRI APC (clone REA975; Miltenyi Biotec). Plasma IgG binding to these activated monocytes was assessed using a goat Fab’2 anti-human-IgG (H + L) AF647 mAb (Southern Biotech, Birmingham, AL, USA). Plasma from the same four healthy donors was recovered following centrifugation of heparinized whole peripheral blood and used for binding assays of GUS, UST, and RZB labeled with AF647 to their autologous activated monocytes. All preparations of cells stained with fluorophore-coupled antibodies were subjected to flow cytometry using the LSR-Fortessa (BD Biosciences); the analysis of the recorded (flow cytometry standard) FCS data was performed using FlowJo 10.7.2 software (Tree Star, Inc.).

### Single-cell transcriptomic analysis

scRNAseq analysis was performed using a previously published protocol from Sachen et al., with patient-level PsO or CD data available at ArrayExpress (E-MTAB-8142) or GEO (GSE134809), respectively (Sachen et al. 2025). In addition, four other scRNAseq datasets were similarly processed for validation, with patient-level PsO data accessible at EGA: EGAS00001005271 or GEO: GSE220116, and patient-level CD data accessible at the Broad Single Cell Portal: SCP1884 or GEO: GSE282122 (Sachen et al. 2025; Liu et al. 2022; Kim et al. 2023; Kong et al. 2023; Thomas et al. 2024). For single-cell analyses and visualization, we utilized SCANPY, a tool for large-scale single-cell gene expression data. In the skin datasets, we retained cells that met the following criteria: total unique molecular identifier counts greater than 1,000, total genes greater than 400, and mitochondrial reads less than 20%. In the tissue datasets from patients with CD, raw counts for each gene were normalized. For analysis of tissues from patients with PsO or CD, cells from lesional/inflamed and nonlesional/uninflamed cells were pooled across all diseased donors and myeloid cell populations were identified by manual consolidation of the cell-type annotations. The percentage of myeloid cells that coexpressed *IL23A* and *FCGR1A (CD64)* gene transcripts per study was represented using Venn diagrams.

### Xenium spatial transcriptomics

Sections (5 µm) of formalin-fixed paraffin-embedded (FFPE) surgical colon resections from patients with chronic UC were placed on the Xenium slide in accordance with the Xenium In Situ for formalin-fixed paraffin-embedded FFPE – Tissue Preparation Guide (CG000578) (10 × Genomics; Pleasanton, CA, USA). The slides were stored at room temperature in a desiccator before proceeding with decrosslinking (Xenium in situ for FFPE – Deparaffinization & Decrosslinking; CG000580). Slides were then hybridized with either the Xenium 5K probe or the IBD in-house custom 480-gene probe panel, including the cell segmentation gene panel, in accordance with the Xenium 5K Prime in situ gene expression with optional Cell Segmentation Staining (CG000760) and Xenium Analyzer (CG000584) protocols. The slides were stained with H & E on the Leica ST5010 autostainer using reagents and methods found in the Xenium In Situ Gene Expression—Post-Xenium Analyzer H & E Staining (CG000613) protocol. Images were captured using the Panoramic 1000 whole-slide scanner (3DHISTECH; Budapest, Hungary). Xenium transcript matrix data were uploaded into the BioTuring Ecosystem SpatialX platform (San Diego, CA, USA). Single-cell type identification and annotation were performed with Seurat R (R Project, Satija Lab at NYGC) and uploaded as metadata into SpatialX, where the samples were selected for evaluation. SpatialX allowed for querying the gene set for the expression of multiple genes in the context of cellular expression. The coexpression of two or more genes was then plotted to obtain the number of cells exhibiting coexpression of the genes of interest.

### Statistical analysis

Statistical analysis was performed using GraphPad Prism 9.5.0 (Boston, MA, USA). An ordinary one-way analysis of variance (ANOVA) was used for statistical comparisons, with Dunnett’s or Sidak’s post hoc test used for multiple comparisons. Statistical comparisons were made with the DPBS-vehicle control, with nominal ****P ≤ 0.0001; ***P ≤ 0.001; **P ≤ 0.01; *P ≤ *0.05*, indicating significance.

## Results

### Anti-mouse IL-23 wild-type and LALA monoclonal antibodies exhibit comparable anti-inflammatory effects in mouse models of colitis

**Fig. 1:**
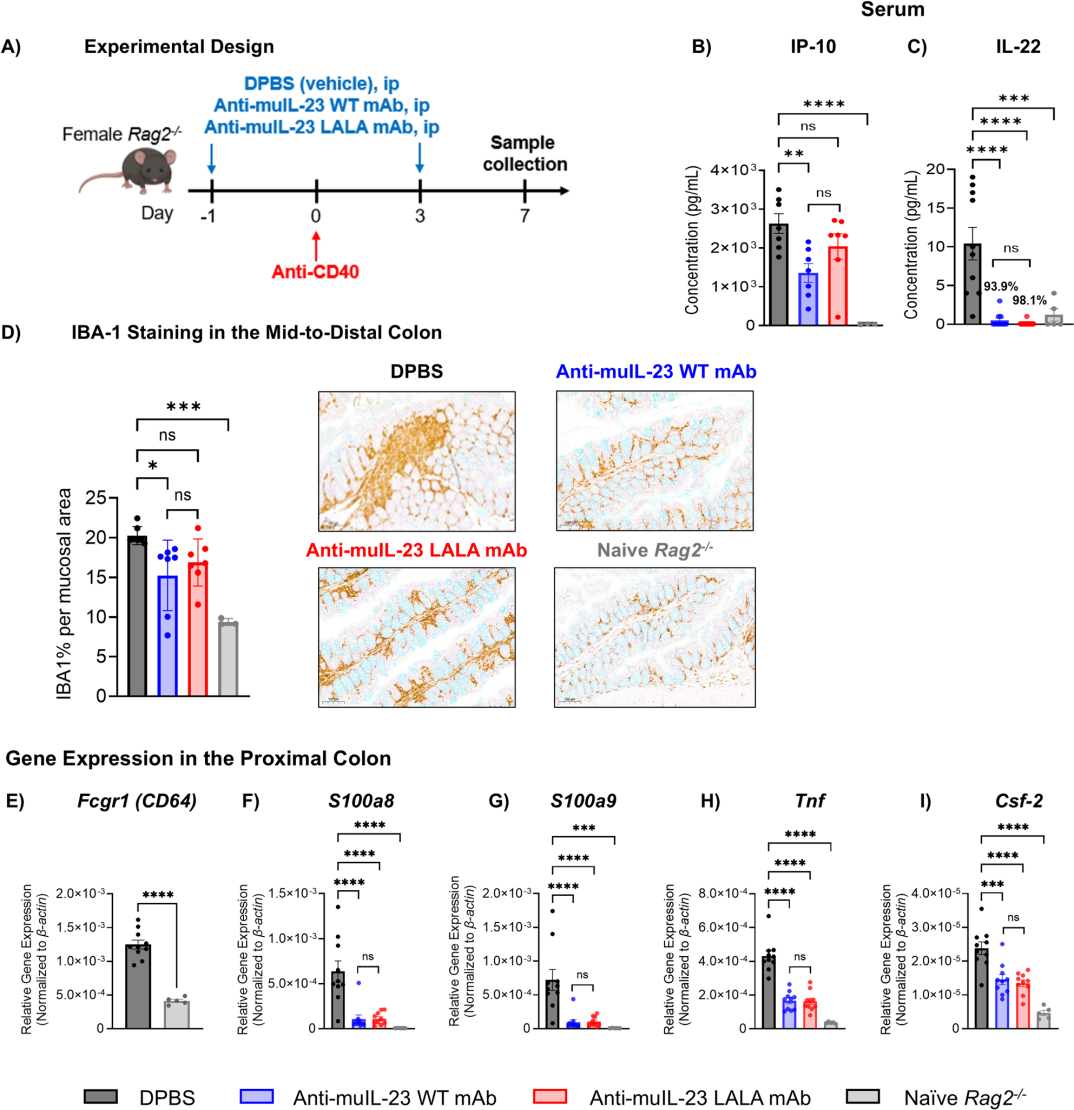
Anti-Mouse IL-23 Monoclonal Antibodies With WT vs LALA Fc Portions Induce Similar Anti-Inflammatory Responses in an Anti-CD40 Rag2-/- Mouse Model of Colitis.ANOVA, analysis of variance; DPBS, Dulbecco’s phosphate-buffered saline; csf-2, colony-stimulating factor 2; IBA-1, ionized calcium‐binding adapter molecule 1; IHC, immunohistochemistry; IL, interleukin; IP-10, Interferon-γ-inducible protein-10; mAb, monoclonal antibody; qPCR, quantitative polymerase chain reaction; Rag, recombination-activating gene; S100 a8, S100 calcium-binding protein a8; S100 a9, S100 calcium-binding protein a9; Tnf, tumor necrosis factor; WT, wild-type. **A** Experimental Design. All subsections have titles in the figure. Female Rag2-/- mice were treated with anti-CD40 on day 0 to induce colitis. Mice received either anti-mouse IL-23 mAb (WT or LALA-modified) or DPBS intraperitoneally on day -1 and day 3. Mice were monitored for 7 days and euthanized on day 7. Serum concentration, **B** IP-10, and (**C**) IL-22, percent of DPBS vehicle control. **D** IHC staining of IBA-1 in the proximal colon. Gene expression analysis of (**E**) Fcgr1, **F** S100a8, **G** S100a9, **H** Tnf, and **I** Csf-2 in the proximal colon was analyzed using qPCR. For panels B-I, tissues were analyzed on day 7. Statistical analysis was performed using a one-way ANOVA, followed by Dunnett’s or Sidak's post hoc test for multiple comparisons. For all comparisons, nominal *P* values indicate significance, with 5-10 mice per group. ****P ≤ .0001; ***P ≤ .001; **P ≤ .01; *P ≤ .05 versus DPBS-vehicle control

We sought to determine the impact of WT and LALA Fc portions on IL-23 neutralization and whether endogenous IgG may have influenced the differences previously reported (Sachen et al. 2025). To assess the anti-inflammatory effects of anti-IL-23 antibodies with differing Fc portions, mAbs bearing either a WT or LALA Fc were evaluated in murine colitis models that either lacked endogenous Ig (anti-CD40-treated *Rag2*^*−/−*^) or retained endogenous Ig (DSS-treated *Il10*^*−/−*^ C57BL/6). To have IL-23 WT and LALA-modified antibodies that bind and inhibit IL-23 with a similar affinity, yet differ primarily in their Fc portion, we utilized the same mouse IgG2a anti-mouse IL-23p19 antibody of high-affinity with or without the Fc mutation LALA (Figure S1) (Mancardi et al. 2008). In *Rag2*^*−/−*^ mice, we utilized the CD40 agonist colitis model and observed an upregulation of FcγRI (CD64) transcripts in the colon compared with naïve mice, supporting the use of the model to evaluate Fc binding and IL-23 neutralization (Fig. 1A, E; S2) (Uhlig et al. 2006). When assessing the systemic anti-inflammatory effects of anti-IL-23 WT and LALA-modified antibodies, we found that the WT antibody preferentially suppressed the IFN-γ response cytokine, IP-10, over the LALA-modified antibody (*P* ≤ 0.01; Fig. 1B). However, the effects of the IL-23 WT antibody were not significantly different from those of the LALA-modified antibody (Fig. 1B). Importantly, when investigating systemic cytokines more closely related to IL-23 signaling, such as IL-22, we observed comparable suppression by both antagonists (*P* ≤ 0.001; Fig. 1C). IHC staining of the colon revealed reduced expression of IBA-1, a marker of macrophages, in *Rag2*^*−/−*^ mice treated with the IL-23 WT antibody compared with DPBS-treated mice (*P* ≤ 0.05, Fig. 1D). Although the IL-23 LALA-modified antibody did not significantly reduce intestinal IBA-1 expression compared with the DPBS vehicle group, there was also no difference compared with the IL-23 WT mAb-treated mice. All other anti-inflammatory effects were comparable between the IL-23 WT mAbs.

Anti-muIL-23 WT and LALA-modified mAbs induced similar suppression of other inflammatory genes, including *S100a8* (protein name: calgranulin A), *S100a9* (protein name: calgranulin B), *Tnf* (protein name: TNF-α), and *Csf-2* (protein name: GM-CSF; *P* ≤ 0.001 or greater; Fig. 1F-I). Despite the lack of endogenous IgGs in the *Rag2*^*−/−*^ mice, which creates the potential for exposure differences due to FcγRI (CD64) binding-mediated disposition, serum concentrations of anti-IL-23 WT and LALA-modified mAbs were similar, approximately 600 µg/mL at the 45 mg/kg dosage used, indicating that results were not attributable to variations in antibody availability in the in vivo mouse model (Figure. S2B). Under conditions of maximal soluble IL-23 neutralization, the data suggest that even when FcγRI (CD64)-dependent IL-23 neutralization by wild-type Fc antibodies may be artificially enhanced, there appears to be no significant anti-inflammatory advantage observed over a LALA-modified anti-IL-23 antibody.

**Fig. 2:**
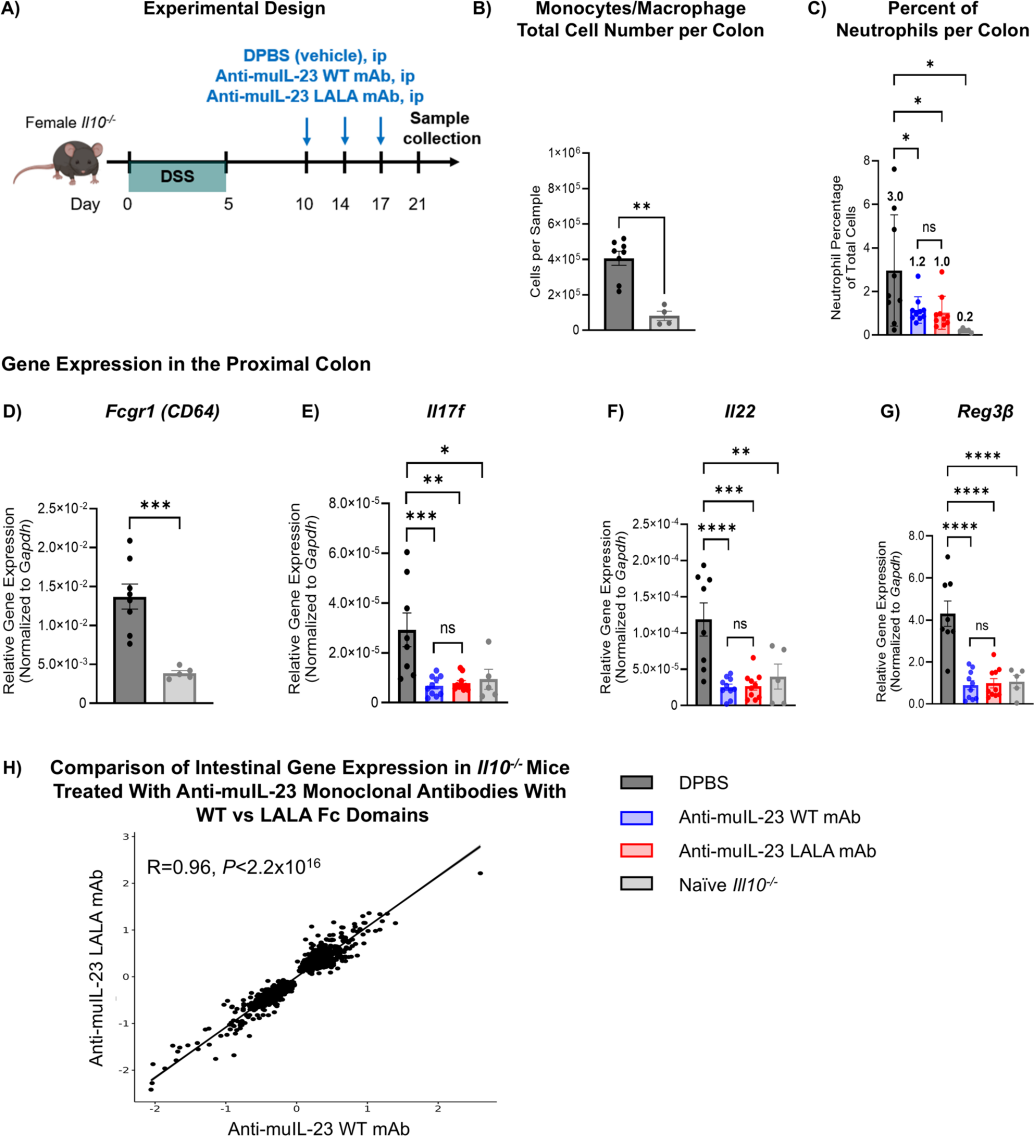
Anti-mouse IL-23 monoclonal antibodies with WT vs LALA Fc portions induce similar anti-inflammatory responses in an *Il10*^*−/−*^ mouse model of colitis. ANOVA, analysis of variance; DSS, dextran sodium sulfate; H and E, hematoxylin and eosin; IL, interleukin; ip, intraperitoneal; DPBS, Dulbecco’s phosphate-buffered saline; mAb, monoclonal antibody; qPCR, quantitative polymerase chain reaction; Reg3β, regenerating islet-derived protein-3β; WT, wild-type. **A** Experimental Design. All subsections have titles in the figure. Female *Il10*^*−/−*^ mice were treated with 3% DSS to induce colitis from days 0–5. Mice received either anti-muIL-23 mAb (WT or LALA-modified) ip on days 10, 14, and 17 and were monitored until day 21. **B** Quantification of colonic FcγRI^+^ (CD64^+^) monocytes/macrophages (CD45^+^/CD11b^+^FcγRI^+^ [CD64^+^]) of DPBS-treated or naïve *Il10*^*−/−*^ mice using flow cytometry analysis. **C** Neutrophil quantification within the distal to midcolon using an algorithm of H&E-stained tissue, with the percentage of neutrophils normalized per colon. Gene expression analysis of (**D**) *Fcgr1,* **E*** Il17f,* **F*** Il22,* and (**G**) *Reg3β* was performed in the proximal colon using qPCR analysis*.* (**H**) Fold change gene correlation analysis in the proximal colon of *Il10*^*−/−*^ mice treated with anti-muIL-23 mAb (WT or LALA-modified) compared with DPBS control, with analysis focused on genes that exhibited significant differential expressions (false discovery rate < 0.05) in at least one of the treatment comparisons. For panel H, statistical analysis was performed using Pearson’s correlation coefficient and regression analysis. For all other panels, statistical analysis was performed using a one-way ANOVA with Sidak’s or Dunnett’s post hoc test for multiple comparisons. For panels B-H, tissues were analyzed on day 21, with 5–10 mice per group. ****P ≤ .0001; ***P ≤ .001; **P ≤ .01; *P ≤ *.05* versus DPBS-vehicle control

We next tested whether an anti-mouse IL-23 mAb with a WT or LALA-modified Fc portion would perform differently in an *Il10*^*−/−*^colitis mouse model, which contained mature B cells and physiologically relevant concentrations of endogenous IgG (Gomes-Santos et al. 2012). *Il10*^*−/−*^ mice were administered 3% DSS in their drinking water from days 0–5, with IL-23 WT or LALA mAbs administered in sterile DPBS three times a week from day 10 to day 21 (days 10, 14, and 17; Fig. 2A). We confirmed that DSS treatment of *Il10*^*−/−*^ mice resulted in an influx of intestinal FcγRI^+^ (CD64^+^) myeloid cells, as determined by flow cytometry and qPCR (Fig. 2B, D). Consistent with findings in the *Rag2*^*−/−*^ model, comparable serum concentrations of anti-IL-23 WT or LALA-modified mAbs were observed in each treatment group at 45 mg/kg, approximately 100 µg/mL (Figure S3). Both anti-IL-23 WT and LALA-modified mAbs significantly reduced the percentage of neutrophils that accumulated in the distal colon, from 3.0% in DPBS-treated controls to 1.2% and 1.0% in IL-23 WT and LALA-modified mAbs-treated groups, respectively (Fig. 2C). Treatment with either of the anti-IL-23 WT or LALA-modified mAbs equally inhibited IL-23-driven inflammatory gene expression in the proximal colon, including suppression of *Il17f, Il22,* and *Reg3β*, compared with DPBS controls (*P* ≤ 0.01; Fig. 2E-G). Moreover, gene correlation analysis revealed that the gene expression profiles of *Il10*^*−/−*^ mice treated with IL-23 WT or LALA mAb were highly similar (*R* = 0.96, *P* ≤ 2.2 × 10^–16^; Fig. 2H). These data demonstrate that in an Ig-competent colitis model, the Fc portion of either IL-23 WT or LALA-modified mAbs did not impact their ability to inhibit DSS-induced intestinal inflammation. Both mAbs demonstrated similar suppression of colonic inflammation in both mouse models of colitis, indicating that Fc-mediated neutralization of IL-23 is not driving the anti-inflammatory efficacy in vivo.

### Endogenous immunoglobulins inhibit binding of anti-IL-23 monoclonal antibodies to FcγRI (CD64)

In the *Il10*^*−/−*^ DSS model, an explanation for the lack of difference between the anti-muIL-23 WT and LALA-modified mAb treatment groups in vivo, may be attributed to high levels of endogenous Ig occupying FcγRI^+^ (CD64^+^) macrophages, which subsequently prevent the targeted disposition of anti-IL-23 WT Fc portion to IL-23-producing cells. Utilizing AF647-labeled IL-23 WT or LALA-modified mAbs, we tested either antibody’s ability to bind to the CHOK1 cell line overexpressing mouse FcγRI (CD64) by flow cytometry in DPBS with 10% FBS ultra-low IgGs or in the presence of 100% C57BL/6 mouse plasma (Figure S1) (Mancardi et al. 2008). Binding inhibition was observed with C57BL/6 plasma, but inhibition was not observed with 100% *Rag2*^*−/−*^ mouse plasma, which lacks endogenous IgGs, indicating that endogenous IgGs in plasma (at approximately 3 mg/mL) effectively block binding to FcγRI (CD64) (Figure S1) (Sarvas et al. 1983).

**Fig. 3:**
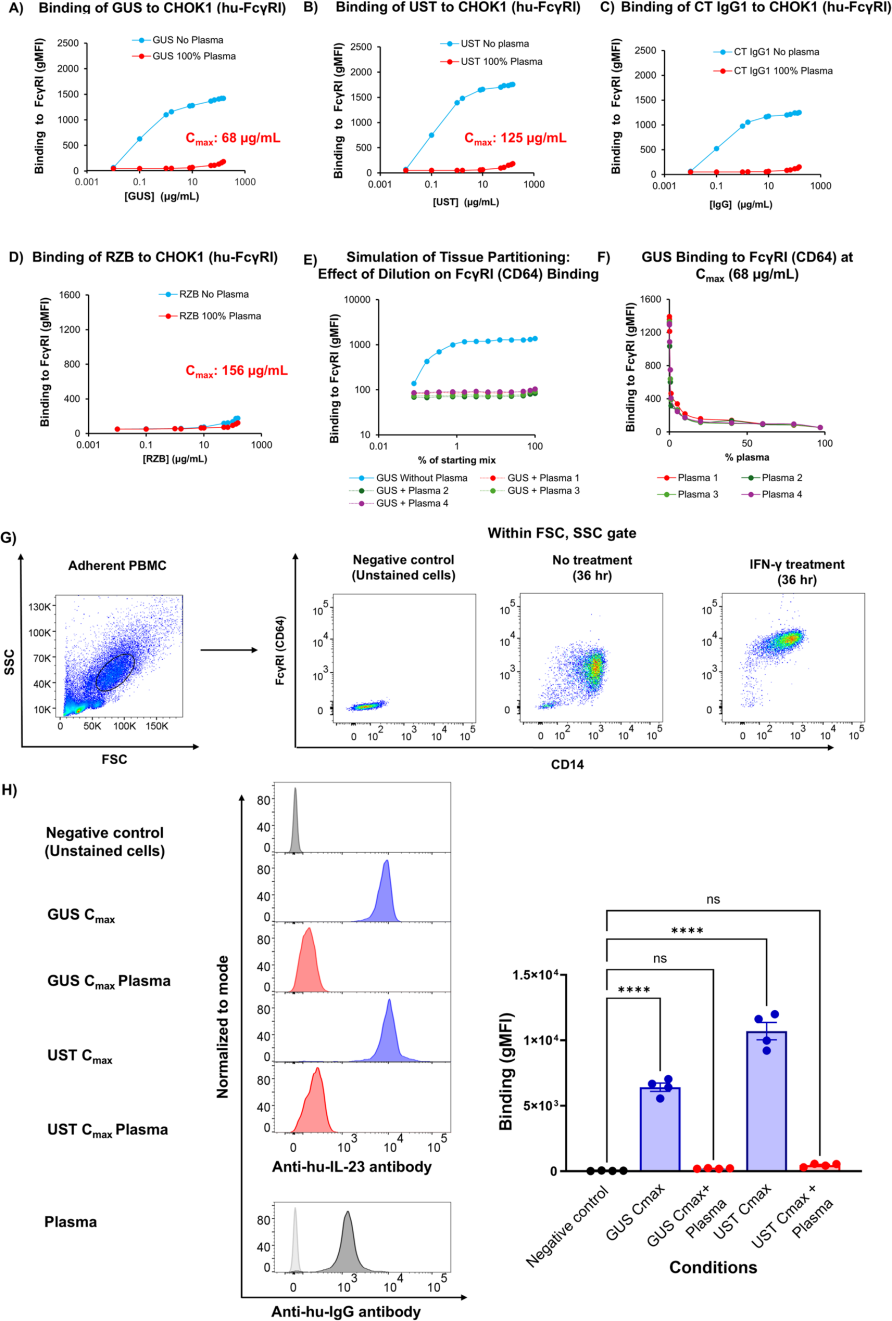
Binding of AF647-labeled GUS, RZB, and UST to FcγRI (CD64) is inhibited using physiological culture conditions. AF647, Alexa Fluor 647; ANOVA, analysis of variance; BSA, bovine serum albumin; CHO-K1, Chinese Hamster Ovary K1 cells; C_max_, maximum concentration; gMFI, geometric mean fluorescence intensity; GUS, guselkumab; hu-FcγRI, human-Fc γ receptor I; IFN-γ, Interferon-γ; IgG1, immunoglobulin G1; mAbs, monoclonal antibodies; PBMC, peripheral blood mononuclear cell; RZB, risankizumab; UST, ustekinumab. Binding assay analysis measuring the binding affinity of AF647-labeled (**A**) GUS, **B** UST, **C** control IgG1, or (**D**) RZB to FcγRI (CD64) on CHOK1 (human-FcγRI) cells, both in the presence and absence of healthy donor plasma. **E** To simulate antibody-tissue partitioning, the effect of dilution on the inhibition of AF647-labeled GUS binding to FcγRI (CD64) was evaluated. “GUS: plasma IgG” at a ratio of 1:70 was utilized as the starting point, and consecutive two-fold dilutions using DPBS supplemented with BSA were applied, generating samples at 100%, 75%, 50%, 25%, 12.5%, 6.25%, 3.12%, 1.66%, 0.83%, 0.41%, 0.2%, 0.1%. **F** Inhibition of GUS binding to FcγRI (CD64) was assessed at the C_max_ concentration in the presence of diluted plasma. **G** IFN-γ (50 ng/mL) was used to activate PBMCs in vitro for 36 h. Flow cytometry analysis of adherent cells showing expression of CD14 and FcγRI (CD64) cell surface markers. **H** Quantification of the binding activity of primary human activated monocytes was measured using the gMFI, in the presence or absence of the AF647-labeled GUS and UST, with or without plasma. Binding activity was also measured in primary human monocytes that were incubated with plasma and stained with a Fab2’-anti-IgG antibody. Negative controls for parts G and H did not include any mAbs (unstained cells). Statistical analysis was performed using one-way ANOVA with Tukey’s post hoc test for multiple comparisons. For all comparisons, nominal *P* values indicate significance, **** P ≤ *.0001* versus negative control

We reproduced these experiments using GUS, RZB, UST, or a control isotype (hu-IgG1) with WT CHOK1 cells or those overexpressing human FcγRI (CD64), CHOK1(hu-FcγRI) (Bruhns et al. 2009). The binding assay was carried out in either DPBS with 10% FBS ultra-low IgG or in full plasma purified from the peripheral blood of healthy donors. While the GUS-AF647, UST-AF647, and control IgG1-AF647 bound to CHOK1(hu-FcγRI) in the absence of plasma, their binding was strongly inhibited in 100% plasma (Fig. 3A-C). RZB-AF647 did not bind to CHOK1(hu-FcγRI), irrespective of the presence or absence of plasma (Fig. 3D).

Endogenous and therapeutic antibody concentrations are often lower in the interstitial space than in plasma due to the limited diffusion of Ig across biological membranes (Ryman and Meibohm 2017). Therefore, it is conceivable that WT Fc therapeutic antibodies may exhibit enhanced binding to FcγRI^+^ (CD64^+^) cells in tissue due to less competition with endogenous Ig. We hypothesized that, in the absence of differences in tissue penetration between therapeutic antibodies and endogenous IgG1, the drug-to-endogenous IgG1 ratio would remain constant, thereby sustaining blockade of GUS binding to FcγRI (CD64). To simulate this antibody tissue partitioning, a two-fold serial dilution of GUS in plasma at C_max_ (denoted by a dotted line in Fig. 3E) or GUS in DPBS at C_max_ (denoted by a solid blue line in Fig. 3E) was carried out, and the binding to CHOK1 (hu-FcγRI) was examined (Fig. 3E**)**. All dilutions of GUS in plasma in a constant ratio sustained inhibition of FcγRI (CD64) binding (Fig. 3E).

Conversely, local increases in the apparent concentration of a biologic can occur in tissues due to reduced amounts of endogenous IgGs. To address this situation, we evaluated the inhibitory effect of the plasma at different concentrations. Blocking with as little as 1% plasma achieved 50% inhibition of FcγRI (CD64) binding to GUS; in comparison, more than 95% inhibition was reached with over 40% plasma, which suggests that even with a local apparent increase in concentration, there is a strong buffering effect of the plasma that prevents the binding of GUS to FcγRI (CD64) (Fig. 3F).

Because CHOK1 cell lines overexpressing hu-FcγRI may not accurately reflect endogenous expression in human primary cells, we further assessed the binding with activated primary human monocytes. PBMCs were isolated by Ficoll gradient, and monocytes were enriched by plastic adhesion. The monocytes were then activated with IFN-γ to upregulate the expression of FcγRI (CD64) (Fig. 3G). GUS, UST, or RZB binding was assessed in the presence or absence of autologous plasma for each of the four donors. Binding of GUS at its maximum bloodstream concentration (C_max_ for patients with IBD: 68 μg/mL) or UST (C_max_ for patients with CD: 125.2 μg/mL) to FcγRI^high^ (CD64^high^) cells was significantly inhibited in the presence of plasma, likely due to the cells’ saturation by their plasma IgGs. (Johnson and Johnson n.d.a, b) Activated monocytes were incubated with plasma and stained with a Fab2’-anti-IgG antibody, demonstrating IgG coating on the cell surface (*P* ≤ 0.0001, Fig. 3H). Similar binding profiles of GUS, UST, and control IgG indicate that GUS and UST do not preferentially bind to FcγRI (CD64) in the presence of their plasma IgGs, supporting the phenomena observed in the overexpressing cells lines. Together, these data support the similarities observed between the WT and LALA-modified IL-23 mAbs in the in vivo preclinical mouse models, which may be due to a lack of targeted accumulation of GUS on FcγRI^+^ (CD64^+^) macrophages.

### Rare per cell colocalization of *IL23A* and *FCGR1A (CD64)* in myeloid cells at the RNA level

**Fig. 4:**
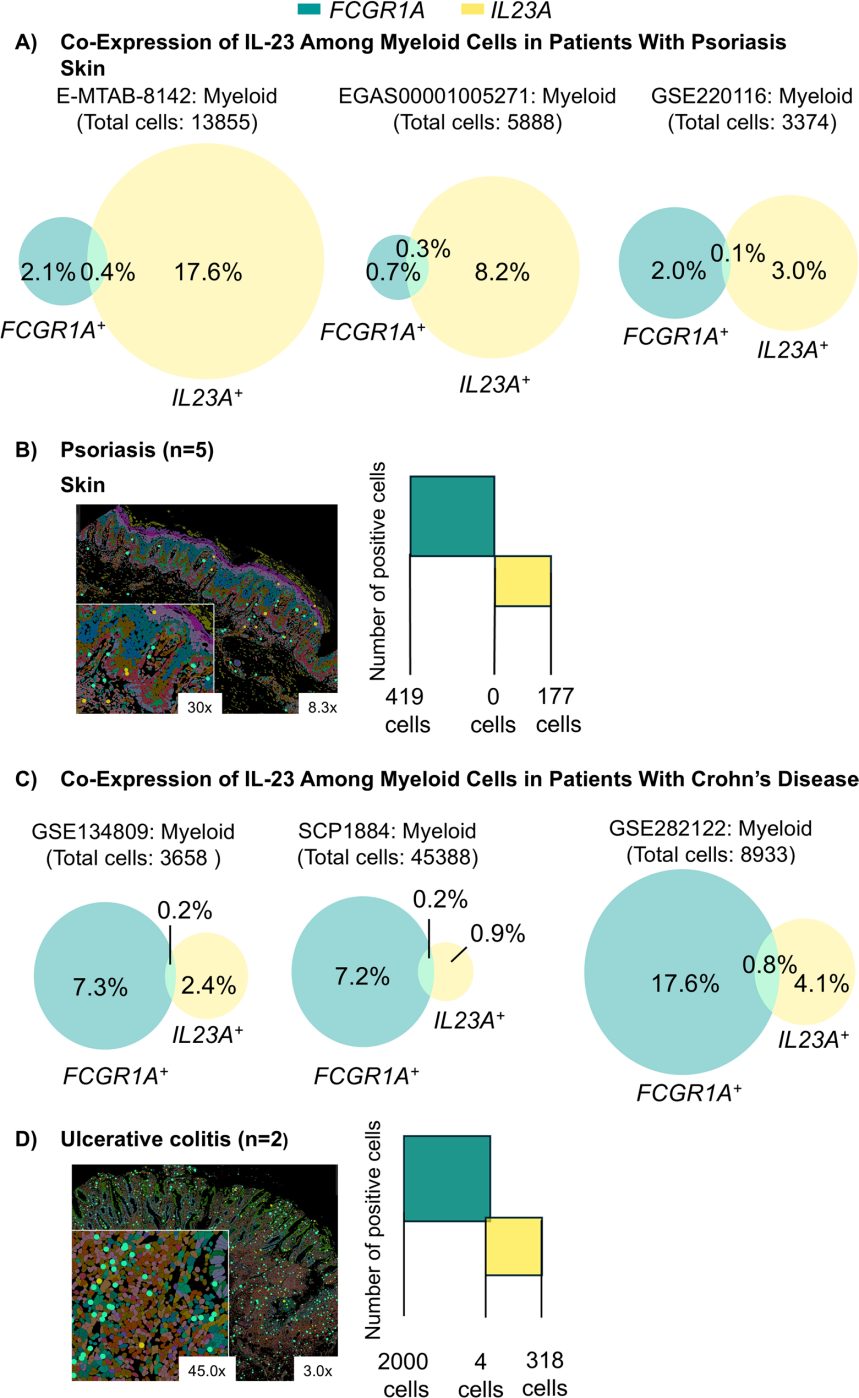
Coexpression of IL23A and FCGR1A (CD64) Transcripts Within Tissues of Patients With Psoriasis or Inflammatory Bowel Disease.FCGR1A, Fc gamma receptor 1A; IL, interleukin; PsO, psoriasis; RNA, ribonucleic acid; scRNAseq, single-cell ribonucleic acid sequencing; UC, ulcerative colitis. **A** scRNAseq Venn diagram analysis was used to quantify the percentage of myeloid cells within skin samples from patients with PsO that expressed either IL23A, FCGR1A (CD64), or both mRNA transcripts in three different databases.(21-24) (**B** and **D**) Representative Xenium spatial transcriptomic images using unsupervised clustering to differentiate cell types based on genetic expression, resulting in cells depicted in various colors in tissues from patients with PsO or UC, displaying FCGR1A (CD64) transcripts as green dots, while IL23A-only transcripts are shown as yellow dots. Insets provide higher magnification. The accompanying box plot illustrates the counts of cells expressing each marker and those coexpressing both IL23A and FCGR1A (CD64) mRNA. **C** scRNAseq and Venn diagram analysis were used to quantify the percentage of myeloid cells within samples from patients with CD that expressed either IL23A, FCGR1A (CD64), or both mRNA transcripts in three different databases. For panels A and C, both lesional/involved and nonlesional/uninvolved tissues from patients with either PsO or CD were examined. See Figure S4 for additional data showing gene expression in dot plots related to panels A and C

Alternatively, anti-inflammatory responses between the anti-IL-23 WT and anti-IL-23 LALA-modified mAb may not differ if IL-23 is not primarily produced by FcγRI^+^ (CD64^+^) cells. Thus, we sought to determine which cells coexpress *IL-23A* and *FCGR1A (CD64)* mRNA transcripts in patient tissue biopsies. Analysis of cells isolated from lesional/involved and nonlesional/uninvolved tissues skin and intestinal samples from patients with PsO or IBD, respectively, revealed that *IL23A* was predominantly expressed by myeloid cells (Figure S4), as previously reported (Sachen et al. 2025). On a per-cell basis, myeloid cells express either *IL23A* (17.6%, 8.2%, 3.0%) or *FCGR1A (CD64)* mRNA transcripts (2.1%, 0.7%, 2.0%) among PsO skin samples in the three databases analyzed (E-MTAB-8142, EGAS00001005271, GSE220116; Fig. 4A). In contrast, cells coexpressing both *IL23A* and *FCGR1A (CD64)* transcripts were detected at low frequencies (0.4%, 0.3%, and 0.1%), suggesting that *FCGR1A*^+^ (*CD64*^+^) cells are unlikely to represent the sole source of *IL23A*.

Xenium spatial transcriptomic imaging was used to further investigate the cellular coexpression of *IL23A* and *FCGR1A (CD64)* transcripts in samples from patients with PsO, preserving the spatial context of the inflamed tissues. While the imaging showed cells that expressed *IL23A* (177 cells) or *FCGR1A* (*CD64,* 419 cells) alone, cells rarely coexpressed both transcripts (Fig. 4B). Similar results were found in tissues from patients with PsA, with as few as 30 cells identified coexpressing both *IL23A* and *FCGR1A (CD64)* transcripts (Figure S5).

Similar to patients with PsO, among patients with CD, myeloid cells were a source of *IL23A* (Fig. 4C). On a per-cell basis, myeloid cells express either *IL23A* (2.4%, 0.9%, 4.1%) or *FCGR1A (CD64)* mRNA transcripts (7.3%, 7.2%, 17.6%) among samples from patients with CD in the three databases analyzed (Fig. 4C). Notably, similar to PsO tissues, little to no coexpression of *IL23A* and *FCGR1A (CD64)* transcripts was observed across the three databases analyzed (0.2%, 0.2%, 0.8%; Fig. 4C).

Similar to patients with PsO, xenium spatial transcriptomic imaging was used to further investigate the coexpression of *IL23A* and *FCGR1A (CD64)* transcripts among patients with IBD, specifically in UC, while preserving the spatial context. While the imaging shows cells that expressed *IL23A* (318 cells) or *FCGR1A* (*CD64*, 2000 cells) mRNA alone, very few (4 cells) were found to coexpress both transcripts (Fig. 4D). These data demonstrate that *IL23A* may be expressed in both FcγRI^+^ (CD64^+^) and FcγRI^−^ (CD64^−^) cells. Even if the scRNAseq underestimates the proportion of FcγRI^+^ (CD64^+^) cells due to a lack of correlation between gene and protein expression, there still remains a large proportion of human cells secreting IL-23 that will not be FcγRI^+^ (CD64^+^).

## Discussion

The present study provides insights into the mechanistic role of the Fc portion in IL-23-neutralizing antibodies, specifically GUS, RZB, and UST, with a focus on their interactions with FcγRI (CD64). Our findings underscore that although WT Fc-neutralizing antibodies possess the potential to engage FcγRs, particularly FcγRI (CD64), these interactions do not contribute to their therapeutic efficacy in vivo. The lack of differences between the mAbs may be attributed to endogenous Ig competing with the WT Fc anti-IL-23 antibody, which binds to FcγRI^+^ (CD64^+^) cells, and the lack of exclusive expression of IL-23 by FcγRI^+^ (CD64^+^) cells.

Both anti-IL-23 WT and LALA Fc antibodies similarly blocked inflammation in both colitis mouse models tested. However, in an Ig-deficient CD40 *Rag2*^*−/−*^ colitis mouse model, an upregulation of inflammatory FcγRI^+^ (CD64^+^) cells was observed, and there was a slight advantage for the Fc-competent antibodies due to the lack of endogenous IgG competition for FcγR binding. Nonetheless, these changes did not result in significant differences between treatment groups, as IL-23-mediated cytokine suppression was equivalent in colonic tissue. When the Fc WT or LALA anti-IL-23 antibodies were assessed in an Ig-competent colitis mouse model, no difference was observed in the suppression of IL-23-mediated cytokines or subsequent inflammation, as assessed by neutrophil recruitment. This suggests that the observation seen in *Rag2*^*−/−*^ mice is Fc-dependent and is lost due to competition by endogenous IgGs, as modification of the Fc portion does not appear to influence colonic inflammation in an Ig-competent mouse model of colitis.

A previous study showed that GUS has the potential to bind FcγRI (CD64) in vitro and in a coculture assay (in the absence of plasma; thus, in a nonphysiological environment). However, FcγRI (CD64)-mediated internalization of GUS displayed potent inhibition of IL-23 signaling, whereas mAbs that lack the capacity to bind CD64 did not exhibit this effect (Sachen et al. 2025). Although binding was detected under nonphysiological conditions (DPBS supplemented with bovine serum albumin [BSA] or culture medium 10% FBS), the introduction of human plasma to simulate the physiological tissue microenvironment resulted in no detectable binding of GUS, RZB, or UST to FcγRI (CD64), precluding all effects dependent on this latter interaction. This finding is likely attributable to competition with endogenous IgG in plasma, as demonstrated with C57BL/6 and *Rag2*^*−/−*^ mouse plasma using IL-23 mAb. The in vitro, plasma-mediated blockade of GUS and UST binding to FcγRI (CD64) at concentrations up to 250 μg/mL is of clinical relevance, considering that GUS and UST reach serum C_max_ of 68 μg/mL and 125.2 μg/mL, respectively, when administered to patients at the highest labeled doses (Johnson and Johnson n.d.a, b). In comparison, the concentration of competing IgG1 in plasma is approximately 5 mg/mL, representing endogenous IgG1 at an excess of ≥ 40 to 70-fold higher in patients with IBD and ≥ 900-fold higher in patients with PsO, relative to the C_max_ of IL-23 mAbs. In patients with IBD, fecal IgG levels ranged from 0.5 to 2.0 mg/g, suggesting that intestinal tissue IgG levels may also be very high (Lin et al. 2018). This supports a key observation from our study, that in physiological settings, FcγRI (CD64) is predominantly occupied by endogenous IgG1, which limits its availability for binding to exogenous biologics (Johnson and Johnson n.d.b, 2025; AbbVie, Inc. n.d.a). This saturation effect suggests that in vivo, therapeutic antibodies such as GUS and UST have minimal opportunity to bind and engage with FcγRI (CD64), thereby reducing the potential impact of FcγRI (CD64) interactions on their therapeutic action. Moreover, diluting plasma did not influence the inhibition of GUS binding to FcγRI (CD64), suggesting that a lower concentration of endogenous IgG, as found in tissues, does not change the binding inhibition of FcγRI (CD64). When evaluated in *Rag2*^*−/−*^ mice, it was surprising that the in vitro potency differences between Fc WT and Fc LALA anti-IL-23 antibodies did not translate into more pronounced differences in vivo, aside from a trend toward reduced inflammatory cell infiltrate and no statistically significant differences. However, this may be due to our decision to maximize IL-23 neutralization in our in vivo systems (45 mg/kg doses). The rationale for maximally inhibiting IL-23 was supported by reports indicating that approved doses of IL-23 antagonist antibodies likely bind and neutralize all unbound IL-23 (Zhang et al. 2019; Proietti et al. 2023).

Moreover, our analysis of scRNAseq datasets highlights that *IL23A* is produced not only by *FCGR1A*^+^ *(CD64)* myeloid cells but also by other cell populations in both PsO and CD tissues. Xenium spatial transcriptomic analysis of inflamed tissues from patients with PsO or UC supports these observations, showing that coexpression of *IL23A* and *FCGR1A (CD64)* transcripts was rarely observed. Our analysis supports other studies that demonstrate that a large source of *IL23A* transcripts in PsO and IBD (UC and CD) is from non-*FCGR1A (CD64)*-bearing cells, such as inflamed epithelial cells (Kim et al. 2024; Li et al. 2018).

Both UST and GUS have an Fc portion that is competent for binding FcγRI (CD64), whereas RZB contains an Fc portion that has reduced binding affinity for FcγRI (CD64). If Fc binding to FcγRI (CD64) can enhance the neutralizing potency of IL-23, one would expect superior clinical outcomes with the FcγRI (CD64)-binding-competent IL-23-neutralizing antibody. While no head-to-head GUS-to-RZB clinical trial has been conducted, RZB and UST have been compared clinically, with RZB demonstrating superior clinical outcomes (Peyrin-Biroulet et al. 2024; Gordon et al. 2018). As previously reported, RZB exhibits superior inhibition of IL-23 compared with UST as demonstrated by enhanced binding affinity, increased inhibition of signal transducer and activator of transcription 3 (STAT3) signaling, reduced T helper 17 (Th17) differentiation, and more pronounced suppression of inflammation in an in vivo mouse model of PsO (Zhou et al. 2021). Therefore, these clinical observations suggest that the driver of clinical efficacy is the binding affinity to IL-23 and the strength of IL-23R signaling inhibition, rather than the FcγRI (CD64) binding competence of an IL-23-neutralizing antibody.

In conclusion, our findings indicate that GUS and RZB do not significantly bind to FcγRI (CD64) under physiological conditions. This lack of significant interaction may contribute to the FcγRI (CD64)-independent therapeutic efficacy of these agents, which appears to be observed irrespective of the presence or absence of the LALA mutation, as documented in previous randomized controlled trials.

## Supplementary Information

Supplementary Material 1.

Supplementary Material 2.

## Data Availability

Data reported in this manuscript are available within the article and its supplementary materials. Additional data may be requested by contacting AbbVie Inc.

## Abbreviations

ADA: Anti-drug antibodies
ANOVA: Analysis of Variance
CD: Crohn’s disease
CHO-K1: Chinese Hamster Ovary K1 cells
DC: Dendritic cell
DGE: Differential gene expression
DPBS: Dulbecco’s phosphate-buffered saline
DSS: Dextran sodium sulfate
ECL: Electrochemiluminescence
Fab: Fragment antigen-binding
Fc: Fragment crystallizable
FcγRI: Fc gamma receptor I (CD64)
GM-CSF: Granulocyte macrophage-colony-stimulating factor
GUS: Guselkumab
IBA-1: Ionized calcium-binding adapter molecule 1
IBD: Inflammatory bowel disease
IFN-γ: Interferon-γ
Ig: Immunoglobulin
IL: Interleukin
ip: Intraperitoneal
IP-10: Interferon-γ-inducible protein-10
mAb: Monoclonal antibody
MSD: Mesoscale discovery
PBMC: Peripheral blood mononuclear cells
PsO: Psoriasis
qPCR: Quantitative polymerase chain reaction
Rag: Recombination-activating gene
RZB: Risankizumab
scRNAseq: Single-cell RNA sequencing
TNF-α: Tumor necrosis factor-α
UC: Ulcerative colitis
UST: Ustekinumab
WT: Wild-type
