# Supplementary material for: CD64 binding potential does not translate into enhanced therapeutic efficacy for anti-IL-23 antibodies under physiologically relevant conditions
Source: Mol Med. 2026 Mar 28;32:70. doi: 10.1186/s10020-026-01462-z (PMC13151102; doi:10.1186/s10020-026-01462-z)
Supplement: Supplementary file 1 — Supplementary Material 1. [file 10020_2026_1462_MOESM1_ESM.docx]

**Table S1. Molecular Information for Anti-Mouse IL-23 Monoclonal Antibodies**

| **Molecule Name** | **Manufacturer Information** | **Molecule ID** |
| --- | --- | --- |
| Anti-mouse IL-23p19-muIgG2a WT | AC-610222 | PR-2318956 |
| Anti-mouse IL-23p19-mu IgG2a LALA/к | AC-610222 | PR-2301064 |

IL-23, Interleukin-23; mAbs, monoclonal antibodies; Rag2, recombination activation gene 2; WT, wild-type.

For both *Rag2^-/-^* and *Il10^-/-^* mouse models, the anti-muP19-muIgG2aWT and anti-muP19-mu IgG2a LALA/к mAbs were the same
