# Supplementary material for: CD64 binding potential does not translate into enhanced therapeutic efficacy for anti-IL-23 antibodies under physiologically relevant conditions
Source: Mol Med. 2026 Mar 28;32:70. doi: 10.1186/s10020-026-01462-z (PMC13151102; doi:10.1186/s10020-026-01462-z)
Supplement: Supplementary file 2 — Supplementary Material 2. [file 10020_2026_1462_MOESM2_ESM.zip › Supplemental material 2/CD64 RZB GUS manuscript_resubmission supp FINAL.docx]

**SUPPLEMENTARY MATERIAL**

**CD64 Binding Potential Does Not Translate Into Enhanced Therapeutic Efficacy for Anti-IL-23 Antibody Under Physiologically Relevant Conditions**

Joel F. Cohen-Solal,^1*^ Calvin S. Pohl,^1*^ Sheila M. Cummings,^1^ Jeremy P. Gygi,^1^ Zhaleh Safikhani,^1^ Brigitte Bartocha,^1^ Christopher D. Buckley,^2-5^ Yongli Dong,^1^ Killian Eyerich,^6,7^ Samuel D. Karsen,^1^ Grace R. Lynch,^1^ Michael Macoritto,^1^ Pierre A. Morisset,^8^ Ornella D. Nelson,^1^ Timothy Radstake,^1^ Florian Rieder,^9^ Jocelyn Rivas,^1^ John P. Savaryn,^8^ Kathleen M. Smith,^1^ Madison Stulir,^1^ Carmin Szynal,^1^ Casey Tylek,^1^ Geertruida M. Veldman,^1^ Laura G. Wasserman,^1^ Susan Westmoreland,^1^ Neha Chaudhary,^1^ Matthew M. Staron^1^

^*^Denotes cofirst authors

^1^AbbVie Inc., Worcester, MA, USA;

^2^Kennedy Institute of Rheumatology, University of Oxford, Oxford, UK;

^3^Translational Gastroenterology & Liver Unit, John Radcliffe Hospital, Headington, Oxford, UK;

^4^Rheumatology Research Group, Institute of Inflammation and Ageing, University of Birmingham, Birmingham, UK;

^5^NIHR Oxford Biomedical Research Centre, Oxford, UK;

^6^Department of Dermatology and Venereology, Medical Center, University of Freiburg, Freiburg, Germany;

^7^Division of Dermatology and Venereology, Department of Medicine Solna, Karolinska Institute, Stockholm, Sweden;

^8^AbbVie Inc. North Chicago, IL, USA;

^9^Department of Inflammation and Immunity, Cleveland Clinic Research; Department of Gastroenterology, Hepatology and Nutrition, Digestive Diseases Institute; Cleveland Clinic Foundation, Cleveland, OH, USA

**Supplementary Methods**

**Quantification of Anti-Mouse IL-23 WT or LALA-Modified Monoclonal Antibodies and Cytokines/Chemokines in Mouse Serum**

A mesoscale discovery (MSD) electrochemiluminescence (ECL) singleplex assay was used to quantify mouse IL-23 IgG2a/к (PR-2318956), mouse IgG2a/к LALA (PR-2301064), IL-22, or IFN-γ-induced protein 10 (IP-10), in mouse serum samples. Total antibody levels were analyzed by employing mouse IL-23 p40-(GGGGS)4-IL-23 p19 (mouse-Tev-Flag-6His) and a goat anti-mouse sulfotag detection reagent. Plate coating and blocking were performed with 3% blocker A in 1x PBS. Capture reagent (0.5 µg/mL) was added, and the mixture was incubated at room temperature on a shaker (600 rpm) for 1 hour. Standard curves were prepared using mouse IL-23 IgG2a/к or mouse IL-23 IgG2a/к/к LALA (0.137-100 µg/mL) with LLOQs of 0.014 µg/mL and 1.014 µg/mL, respectively. Dilution of samples ranged from 7,000x to 28,000x. Following a washing step, detection reagent (0.25 µg/mL) was added and incubated at room temperature on a shaker for 1 hour. In the final step, a 1x read buffer was added before reading on an MSD QuickPlex SQ 120 instrument (Rockville, MD, USA). All reagents for the MSD ECL assay were purchased from MSD (Rockville, MD, USA).

MSD standard curve fitting (4PL) and data evaluation were performed using XLfit4 software (v5.5.0.5). Calibration curves were plotted from MSD luminescence units versus theoretical standard concentrations. Plates were considered valid when at least two-thirds of the QC were within 30% of the expected values. Values that were below the quantitation limit were treated as 0 when computing mean concentrations and pharmacokinetic parameters. The same protocol was used to quantify anti-IL-23 WT or LALA-modified mAbs in mouse *Il10^-/-^* serum samples, except that the samples were diluted from 10,000x to 200,000x.

| **Molecule Name** | **Manufacturer Information** | **Molecule ID** |
| --- | --- | --- |
| Anti-mouse IL-23p19-muIgG2a WT | AC-610222 | PR-2318956 |
| Anti-mouse IL-23p19-mu IgG2a LALA/к | AC-610222 | PR-2301064 |

**Table S1. Molecular Information for Anti-Mouse IL-23 Monoclonal Antibodies**

IL-23, Interleukin-23; mAbs, monoclonal antibodies; Rag2, recombination activation gene 2; WT, wild-type.

For both *Rag2^-/-^* and *Il10^-/-^* mouse models, the anti-muP19-muIgG2aWT and anti-muP19-mu IgG2a LALA/к mAbs were the same

**Figure S1. Characterization of Anti-Mouse IgG2a Antibodies Targeting IL-23 With Wild Type (WT) or LALA Mutated Fc Portion.**

gMFI, geometric mean fluorescence intensity; IL, interleukin; NT, non-transfected; SPR, surface plasmon resonance; WT, wild-type.

A) SPR analysis for anti-muIL-23 IgG2a/к (Fc WT) or mu-IgG2a LALA/к (Fc L234A, L235A mutated). B) Phenotyping of the CHOK1 (mu-FcγRI) cell line (Pasteur Institute) with the monoclonal anti-mouse FcγRI (CD64) antibody.(1) Negative control was a nontransfected cell line, CHOK1(NT). C) Binding assay measuring the binding to CHOK1 (mu-FcγRI) of AF647-labeled anti-mu IL-23 IgG2a/к or mu-IgG2a/к LALA at concentrations ranging from 0.01 mg/mL to 750 mg/mL in the presence or absence of C57BL/6 or C57BL/6 *Rag2^-/-^* mouse plasma. Binding activity was quantified using the gMFI.

**Figure S2. Validation of Anti-IL-23 wild-type (WT) or LALA-Modified Monoclonal Antibody Treatment in a Preclinical *Rag2^-/-^* Colitis Mouse Model.**

IL, interleukin; mAb, monoclonal antibody; *Rag*, recombination-activating gene; WT, wild-type.

A) Percent change in body weight in *Rag2^-/-^* mice treated with either anti-IL-23 mAb (WT or LALA-modified), DPBS, or naïve *Rag2^-/-^* mice, from days -1 to 7. B) Serum analysis of anti-IL-23 WT and LALA-modified mAbs concentrations on day 7.

**Figure S3. Validation of Anti-Mouse IL-23 WT and LALA-Modified Monoclonal Antibody Treatment in a Preclinical *Il10^-/-^* Colitis Mouse Model.**

FcγRI, Fc γ receptor I; IL, interleukin; DPBS, Dulbecco’s phosphate-buffered saline; mAb, monoclonal antibody; WT, wild-type.

A) Percent change in body weight in *Il10^-/-^* mice treated with either anti-IL-23 mAb (WT or LALA-modified), DPBS, or naïve *Il10^-/-^* mice, from days 0-21. B) Serum concentration of WT or LALA-modified IL-23 mAbs in *Il10^-/-^* mice on day 21.

**Figure S4. Expression of *FCGR1A (CD64) and IL23A* Transcripts in Patients With Psoriasis or Crohn’s disease.**

Activated DC, activated dendritic cell; CD, Crohn’s disease; Inf. mac, inflammatory macrophage; DC, dendritic cell; DC1, type 1 dendritic cell; DC2, type 2 dendritic cell; migDC, migratory dendritic cell; macro, macrophage; mono,mac, monocyte-derived macrophage; moDC, monocyte-derived dendritic cell; pDC, plasmacytoid dendritic cell; PsO, psoriasis; resident mac, resident macrophage.

This figure was reproduced based on previously published data.(2) Single-cell RNA sequencing was used to quantify *IL23A* and *FCGR1A (CD64)* gene transcripts. Cell types are indicated to the left of dot plots; the number of cells for each type in the data used in these analyses is shown on the right. Panels A and B show the percentage of cells in the myeloid population from lesional/involved and nonlesional/uninvolved tissues, respectively, in patients with A) PsO or B) CD.

**Figure S5. Coexpression of *IL23A* and *FCGR1A*** **(CD64) Transcripts Within Inflamed Tissues of Patients With Psoriatic Arthritis.**

Representative Xenium spatial transcriptomic image and coexpression quantification in which *FCGR1A* transcripts are represented by green dots and *IL23A* are represented by yellow dots. Insets provide higher magnification, highlighting the spatial distribution of transcripts. Accompanying box plots illustrate the counts of cells expressing each marker and those coexpressing both *IL23A* and *FCGR1A (CD64)* mRNA.

**References**

1. Mancardi DA, Iannascoli B, Hoos S*, et al.* FcgammaRIV is a mouse IgE receptor that resembles macrophage FcepsilonRI in humans and promotes IgE-induced lung inflammation. *J Clin Invest*, 2008. **118:** 3738-3750.

2. Sachen KL, Hammaker D, Sarabia I*, et al.* Guselkumab binding to CD64+ IL-23–producing myeloid cells enhances potency for neutralizing IL-23 signaling. *Front Immunol*, 2025. **16**.
