# Supplementary figures and images for: CD64 binding potential does not translate into enhanced therapeutic efficacy for anti-IL-23 antibodies under physiologically relevant conditions
Source: Mol Med. 2026 Mar 28;32:70. doi: 10.1186/s10020-026-01462-z (PMC13151102; doi:10.1186/s10020-026-01462-z)

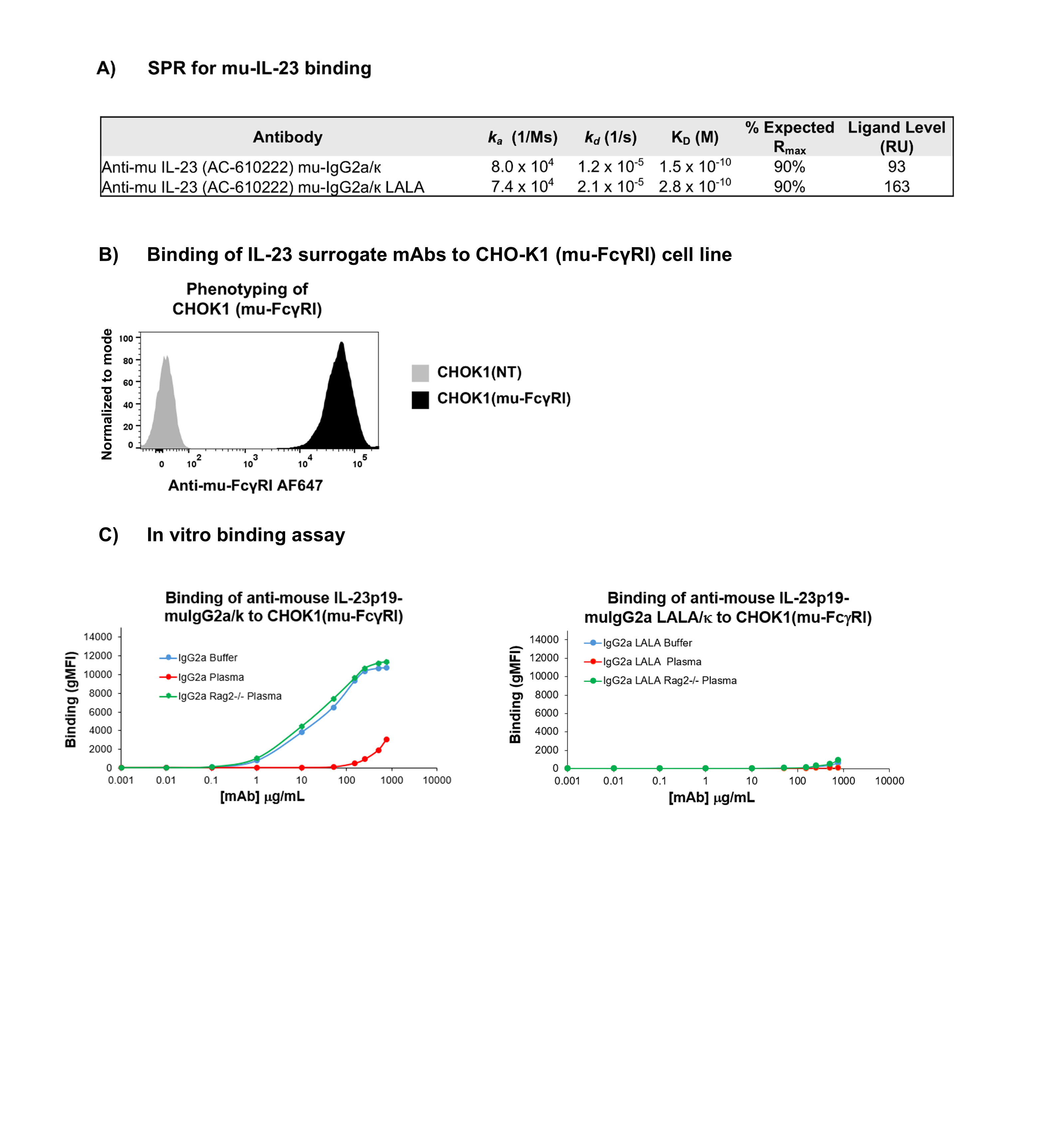

Supplement: Supplementary file 2 — Supplementary Material 2. [file 10020_2026_1462_MOESM2_ESM.zip › Supplemental material 2/Figure S1.TIF]

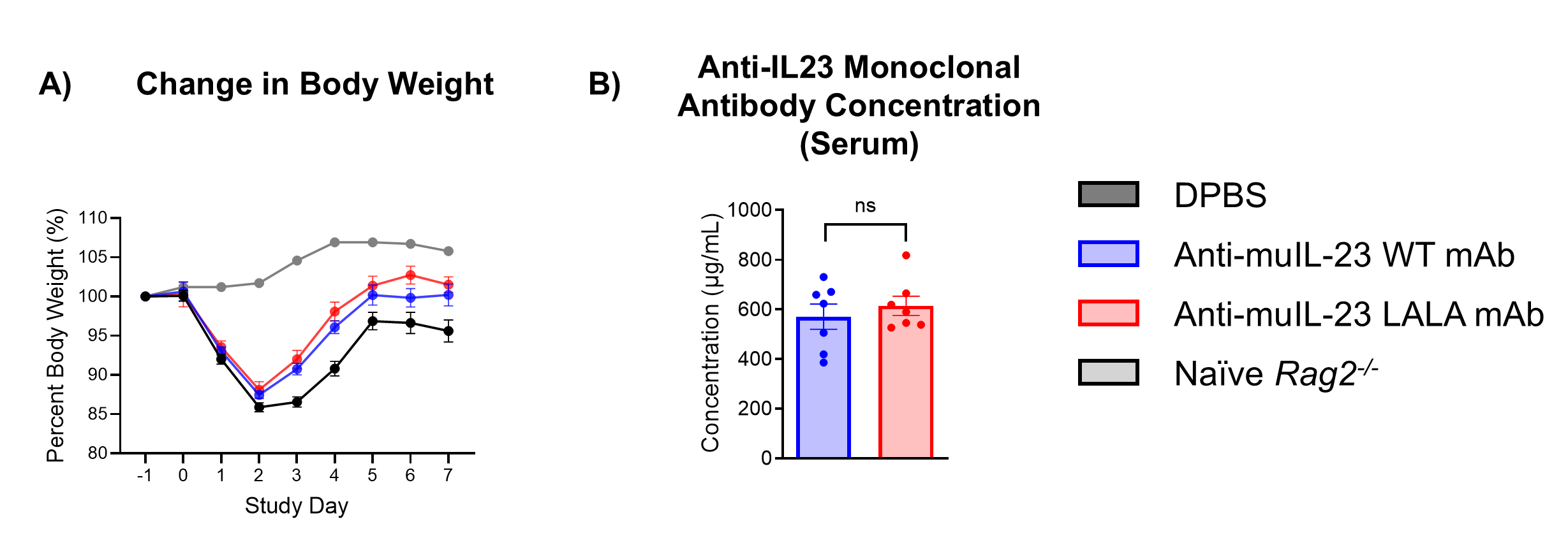

Supplement: Supplementary file 2 — Supplementary Material 2. [file 10020_2026_1462_MOESM2_ESM.zip › Supplemental material 2/Figure S2.TIF]

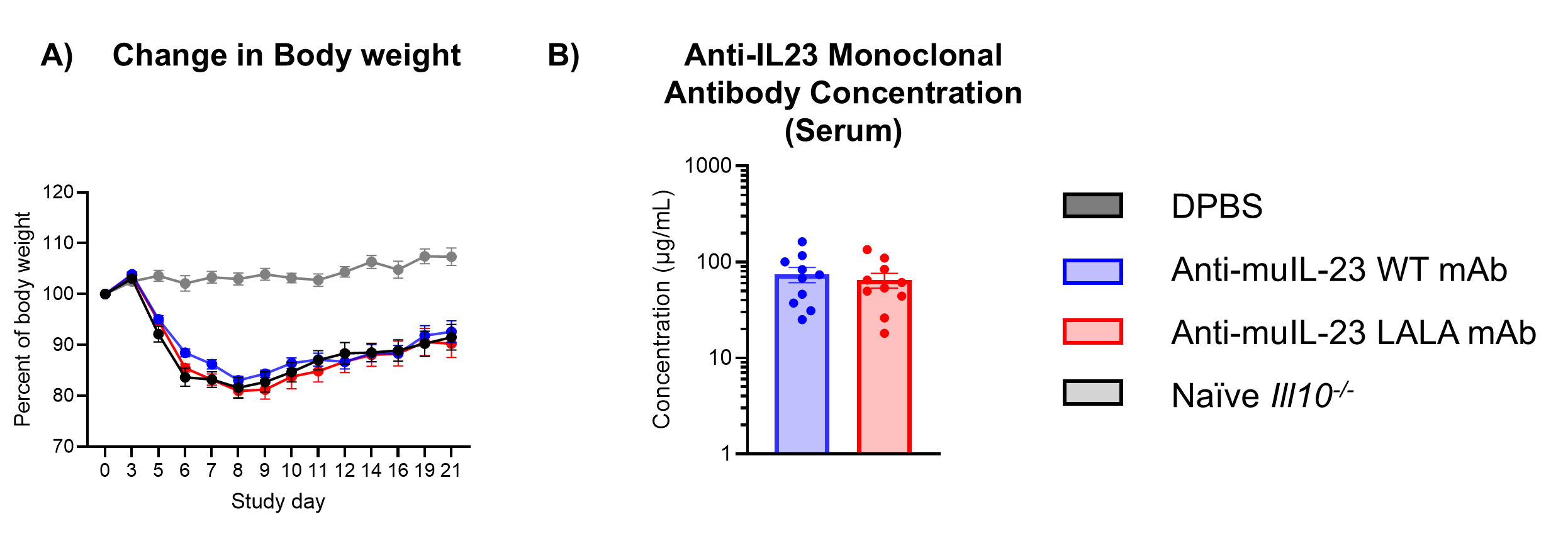

Supplement: Supplementary file 2 — Supplementary Material 2. [file 10020_2026_1462_MOESM2_ESM.zip › Supplemental material 2/Figure S3.TIF]

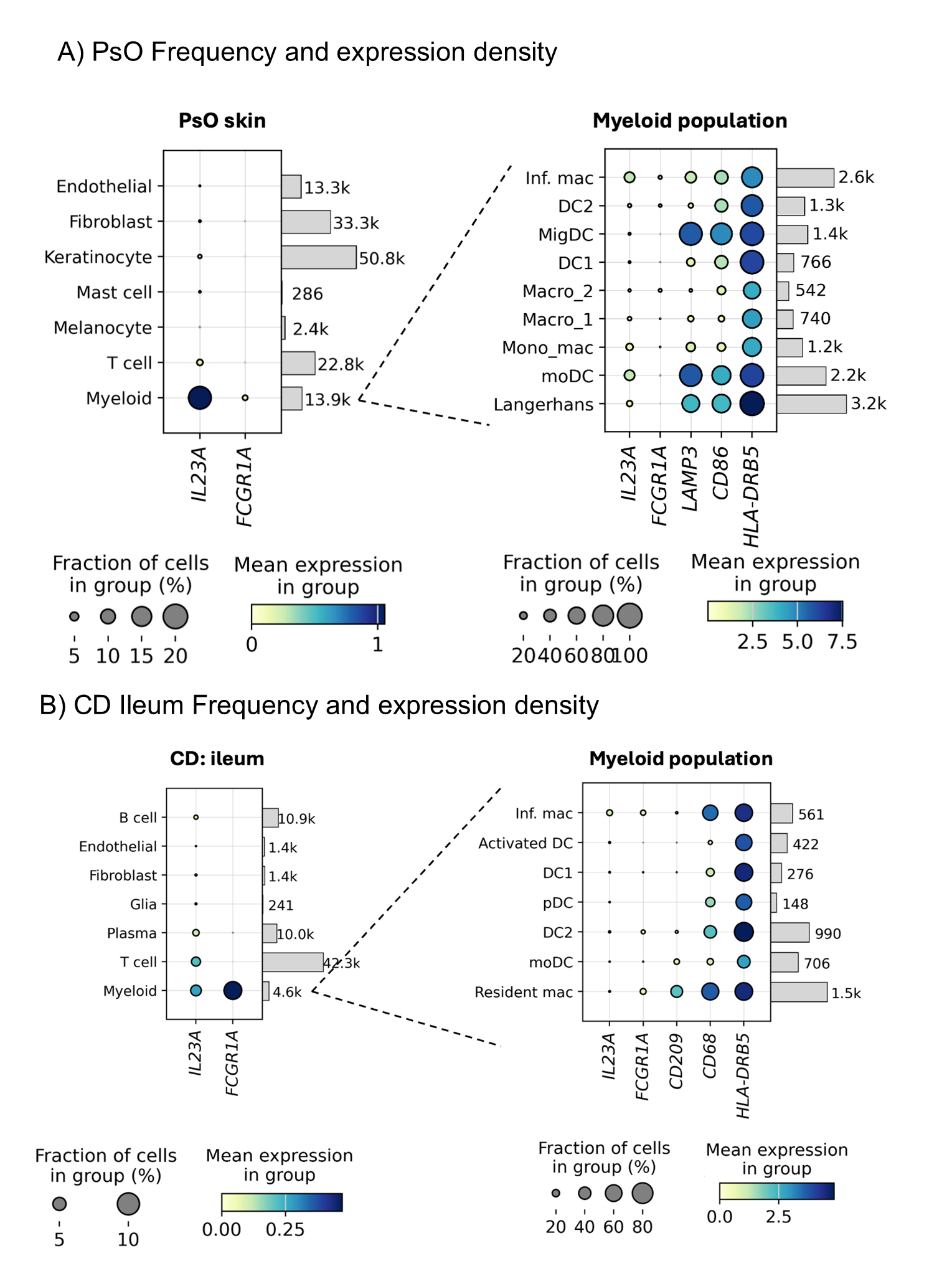

Supplement: Supplementary file 2 — Supplementary Material 2. [file 10020_2026_1462_MOESM2_ESM.zip › Supplemental material 2/Figure S4.TIF]

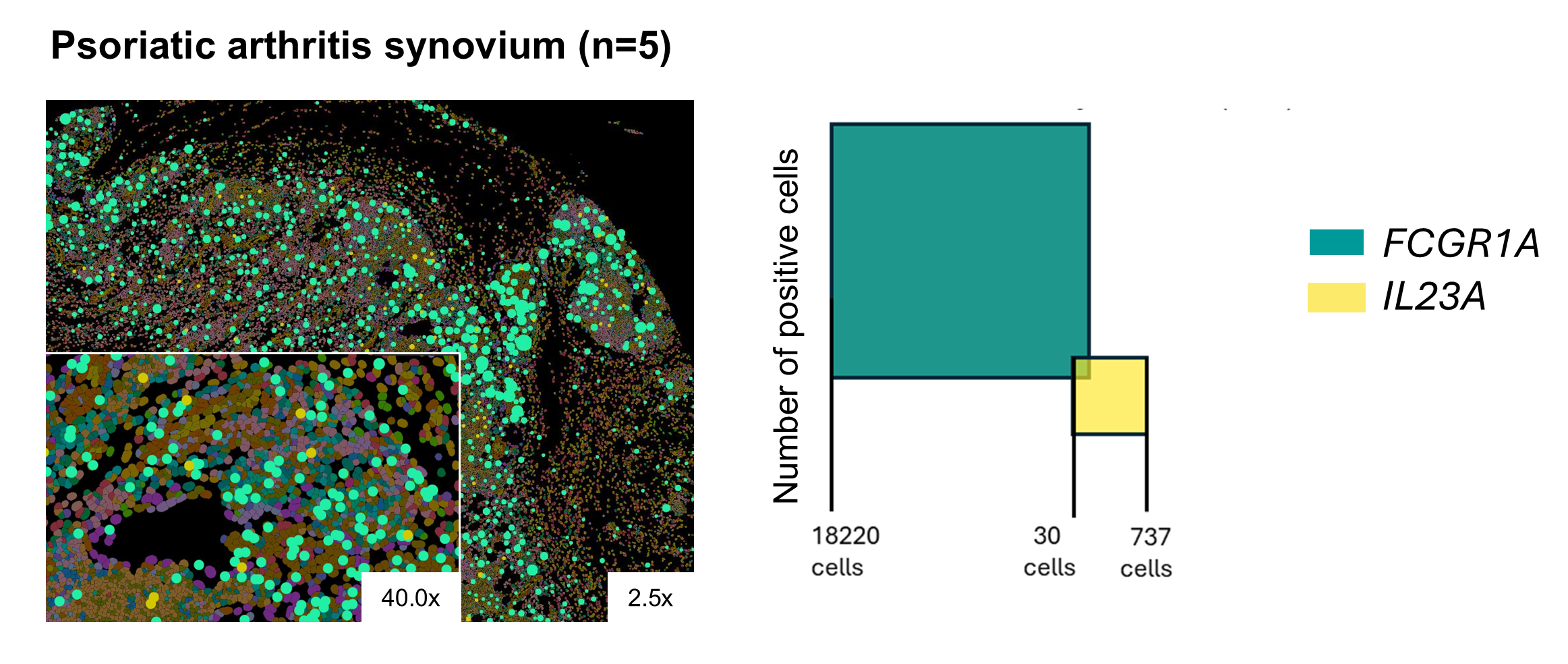

Supplement: Supplementary file 2 — Supplementary Material 2. [file 10020_2026_1462_MOESM2_ESM.zip › Supplemental material 2/Figure S5.TIF]
